# Transcriptome and Metabolome Analyses Reveal the Accumulation Mechanism of Carbohydrates During *Paeonia ostii* Seed Development

**DOI:** 10.3390/biom16010017

**Published:** 2025-12-22

**Authors:** Zhen Li, Siyuan Lv, Yumeng Liu, Mengtian Cao, Hengjia Zhang, Qing Hao

**Affiliations:** 1 College of Agriculture and Biology, Liaocheng University, Liaocheng 252000, China; zhenli@lcu.edu.cn (Z.L.); 2022404343@stu.lcu.edu.cn (S.L.); 2023402391@stu.lcu.edu.cn (Y.L.); 2023402384@stu.lcu.edu.cn (M.C.); 2 College of Landscape Architecture and Forestry, Qingdao Agricultural University, Qingdao 266109, China

**Keywords:** *Paeonia ostii*, seed development, carbohydrate, starch and sucrose metabolism, sugar transporter

## Abstract

Carbohydrate accumulation during seed development directly influences the oil yield and quality of oilseed plants. To clarify the metabolic and molecular mechanisms underlying this process, we examined seed morphology, metabolome, and transcriptome profiles of *Paeonia ostii*, a representative oil tree peony, using molecular biology, bioinformatics, and GC-MS techniques. Seeds expanded rapidly and reached their maximum size at 60 days after pollination, coinciding with increased starch staining intensity. Carbohydrate metabolic patterns indicated the conversion of monosaccharides such as glucose, fructose, and inositol into disaccharides like sucrose and into polysaccharides, including starch, raffinose, cellulose, and hemicellulose. Differentially accumulated carbohydrates and associated genes were enriched in the starch and sucrose metabolism and ABC transporter pathways. We constructed a potential regulatory network comprising genes encoding sugar transporters (SWEET, SUS), glycosyl hydrolases, and transcription factors (NF-Y, MYB, LBD, Dof, and B3), which likely play essential roles in carbohydrate deposition and seed development. Therefore, this study clarifies the metabolic and molecular processes governing carbohydrate accumulation in developing seeds and provides a basis for breeding high-yield, high-quality oil tree peony varieties.

## 1. Introduction

Tree peony (*Paeonia* section *Moutan* DC.) is a perennial deciduous shrub endemic to China with considerable ecological, ornamental, and economic value. *P. ostii* has emerged as an important woody oilseed species, and its seed oil has been approved for human consumption since 2011. Tree peony seed oil contains over 90% unsaturated fatty acids (UFAs), with alpha-linolenic acid (ALA) accounting for ~45% of the total fatty acid content [1]. It is also rich in bioactive compounds such as vitamin E and flavonoids [2]. These attributes confer multiple health benefits, including antioxidant and anti-inflammatory activities, reduced risk of coronary heart disease, and support for neural development. Consequently, peony seed oil holds strong potential for applications in the food, health, and medical sectors. However, current production remains insufficient. Low seed yield and variable quality restrict supply and lead to high market prices.

Seed development in oilseed crops is central to determining both yield and oil quality. The transition from embryo formation to seed maturity encompasses three major stages: the rapid cell division stage, the seed filling stage, and the dehydration and maturation stage [3]. Although most studies have examined fatty acid biosynthesis during seed maturation [1], far fewer have addressed carbohydrate accumulation during the grain-filling phase. However, metabolic activity during seed filling directly influences the availability of substrates required for later fatty acid synthesis. Metabolites such as sucrose and phosphoglycerate act as key carbon precursors for lipid biosynthesis [4]. Increasing evidence indicates that carbohydrate accumulation during early development and grain filling is a major determinant of seed size and yield [5]. Research on oilseed species such as *Linum usitatissimum* and *Camellia drupifera* further underscores the critical role of carbohydrate metabolism in regulating seed oil yield [6,7]. Multi-omics analyses have also identified elevated starch metabolism and glycolytic enzyme activity as potential contributors to the higher oil content of *Brassica napus* relative to other crops [8].

Carbohydrate accumulation in seeds depends on coordinated source–flow–sink activity. Metabolic reactions within seeds are primarily shaped by flow intensity and sink activity, both of which are closely associated with sugar transporters and enzymes involved in carbohydrate metabolism [9]. In rice, *OsSUS1* and *OsSUS3* increase starch content and thereby influence seed size [10,11]. Similarly, overexpression of a potato *SUS* gene in cotton elevates fructose and glucose levels, fiber length, and fresh seed weight in transgenic plants [12]. Additionally, enzymes associated with structural polysaccharide remodeling, including xylanase XYL1, UDP-glucose 4-aminopentadienyl synthase ST1, and hexokinase OsHXK3, also act as positive regulators of seed size [13,14,15]. Sugar transporters essential for seed development include sucrose transporter (SUC/SUT), monosaccharide transporter (MST), and Sugars Will Eventually be Exported Transporter (SWEET). SUC/SUT mainly mediate long-distance sucrose transport from the phloem to developing seeds. In Arabidopsis, the phloem transporter AtSUC2 delivers sucrose to seeds, thereby enhancing sugar metabolism and increasing seed size in overexpressing lines [16]. *AtSUC5* functions during early seed development, and mutants exhibit a strong but transient reduction in fatty acid concentrations [17]. In *Brassica napus*, *BnA7.SUT1* positively regulates pod size and seed weight, with markedly increased expression during grain filling [18]. SWEET proteins also play key roles in regulating seed development and yield. Members of this family in Arabidopsis, soybean, and rice positively influence endosperm development and seed size [19]. In Arabidopsis, *SWEET11*, *SWEET12*, and *SWEET15* control carbohydrate allocation from the seed coat to the embryo [20]. In soybean, *GmSWEET10a*, *GmSWEET10b*, and *GmSWEET15* increase seed size and oil content by enhancing carbohydrate accumulation [21,22]. In rice, OsSWEET4 positively regulates seed filling rate and seed size by interacting with the MATE (Multidrug and Toxic Compound Extrusion) transporter GFD1 (Grain Filling Duration 1) [23].

Transcription factors play a central role in regulating carbohydrate metabolism and transport in seeds by coordinately modulating their target genes. For example, OsNAC127 and OsNAC129 regulate rice seed filling by activating *OsMST6* and *OsSWEET4* [24]. In maize, ZmNAC128 and ZmNAC130 enhance the expression of genes encoding granule-bound starch synthase (*ZmGBSS1*), starch synthases (*ZmSS1* and *ZmSSIIa*), sucrose synthase (*ZmSUS1*), and sugar transporters (*ZmSWEET4c* and *ZmSUGCAR1*), thereby promoting sugar transport and starch synthesis in the endosperm [25]. In Arabidopsis, the Dof transcription factor AtCOG1 increases seed and plant size by enhancing photosynthetic capacity and sugar accumulation [26]. Members of the B3 transcription factor family also participate in seed carbohydrate accumulation and fatty acid biosynthesis. The B3 transcription factor ZmABI19 functions upstream of *ZmbZIP22*, *ZmNAC130*, and *ZmSWEET4c* and is essential for endosperm and embryo development [27]. LEC2, ABI3, FUS3, and the NF-YB transcription factor LEC1 form the core regulatory module LAFL involved in seed development [28]. Within this module, LEC2 acts as the primary regulator of seed storage deposition, while FUS3 serves as a central modulator. In soybean, GmLEC2a activates downstream targets including *GmLEC1*, *GmFUS3*, *GmABI3*, *GmDof11*, *GmWRI1*, and *GmOLE1*, thereby promoting the expression of genes required for carbohydrate accumulation and lipid synthesis during seed storage reserve formation [29]. In both Arabidopsis and cucumber, FUS3 also influences seed yield by activating genes related to carbohydrate metabolism and embryo development [30,31].

Carbohydrate metabolism and transport strongly affect nutrient accumulation in oilseed plants because these processes determine the supply of free sugars that support seed development and oil biosynthesis [32]. However, the metabolic and molecular mechanisms governing carbohydrate accumulation in oil tree peony seeds remain largely unknown. To elucidate the metabolic and molecular mechanisms underlying carbohydrate accumulation of the seed of oil tree peony, this study performed phenotypic, transcriptomic, and carbohydrate-targeted metabolomic analysis on the seed at different developmental stages of *P. ostii*, which is the most widely cultivated oil tree peony. The results revealed the dynamic patterns of carbohydrate metabolism and associated gene expression and clarified key metabolic pathways and genes involved in carbohydrate metabolism and transport. Therefore, these findings deepen our understanding of nutrient accumulation and yield formation in tree peony seeds and may provide valuable insights for the synthetic biology of fatty acid production.

## 2. Materials and Methods

### 2.1. Plant Materials

*P. ostii* plants used in this study were grown in the experimental garden in Heze, Shandong Province. Seed samples at four developmental stages (1 d, 30 d, 60 d, and 80 d after pollination, designated S1, S2, S3, and S4, respectively) were collected with permission from three trees bearing more than 20 flowers each, with each tree serving as one biological replicate. To account for variability within a single plant, multiple seed pods and seeds were collected from each plant and pooled to form one composite sample per stage. Flowers were hand-pollinated with *P. ostii* pollen to ensure seed set. All seeds were immediately frozen in liquid nitrogen and stored at −80 °C.

### 2.2. Determination of Seed Morphology and Histological Staining

Thirty seeds from each stage were randomly selected to measure fresh and dry weights. Another fifteen seeds were randomly selected to determine length, width, and height. For starch staining, seeds were cut longitudinally and treated with iodine solution (4% potassium iodide, 1% iodine) for 2 min.

### 2.3. Sample Preparation and Analysis for Carbohydrate-Targeted Metabolomic

Seeds for each stage stored at −80 °C were homogenized in a mixer mill with a zirconia bead (1.5 min at 30 Hz). Approximately 20 mg of lyophilized powder was extracted in 500 μL methanol–isopropanol–distilled water (3:3:2, *v*/*v*/*v*), vortexed for 3 min, and sonicated at 4 °C for 30 min. The samples were centrifuged at 12,000 rpm for 3 min at 4 °C, after which 50 μL of supernatant was transferred to a new tube and mixed with 20 μL D-Ribose and Adonitol (1000 μg/mL) as internal standards. The mixture was freeze-dried under nitrogen gas and subjected to derivatization. Dried extracts were incubated with 100 μL methoxyamine hydrochloride in pyridine (15 mg/mL) at 37 °C for 2 h, followed by the addition of 100 μL MSTFA and incubation at 37 °C for 30 min. A 50 μL aliquot of the reaction mixture was combined with 1 mL hexane and filtered through a 0.22 μm membrane into chromatographic vials [33]. Pooled samples served as quality control (QC) samples for every batch of 10 samples.

Sugar profiling was performed using an Agilent 8890 gas chromatograph coupled to a 5977B mass spectrometer equipped with a DB-5MS column (30 m × 0.25 mm i.d. × 0.25 μm film thickness; J&W Scientific, Folsom, CA, USA) [34]. Mass spectrometric analysis was performed in selected ion monitoring (SIM) mode. Raw data from the GC-MS platform were processed using Agilent MassHunter software (MultiQuant3.0.3, SCIEX, Redwood City, CA, USA) for peak extraction and alignment. Carbohydrate metabolites were identified and quantified by matching against Metware’s database. Qualitative identification of compounds relied on the correspondence of their characteristic fragment ions and chromatographic retention times with those of authentic standards. Calibration curves were generated for each target metabolite by plotting the concentration ratio (analyte vs. internal standard) against the corresponding peak area ratio across a series of standard concentrations (0.001 μg/mL, 0.002 μg/mL, 0.005 μg/mL, 0.01 μg/mL, 0.02 μg/mL, 0.05 μg/mL, 0.1 μg/mL, 0.2 μg/mL, 0.5 μg/mL, 1 μg/mL, 2 μg/mL, 5 μg/mL, 10 μg/mL, 20 μg/mL, and 50 μg/mL) (Table S1). Final metabolite concentrations in samples were determined by applying the sample’s analyte/internal standard peak area ratio to the fitted curve function.

Seeds stored at −80 °C were also analyzed for starch, cellulose, and hemicellulose contents using commercial detection kits (item numbers G0507W, G0519, and G0527; Grace Biotechnology Co., Ltd., Suzhou, China). Briefly, starch was hydrolyzed to glucose by acid hydrolysis. The glucose content was then quantified using the anthrone colorimetric method. The resulting starch content was calculated based on the glucose measurement. The contents of cellulose and hemicellulose were determined as follows: Soluble sugars and starch were first removed from the plant material. The insoluble residue was then hydrolyzed and analyzed for glucose and xylose content, with subsequent calculation of cellulose (from glucose) and hemicellulose (from xylose) content based on stoichiometric conversion factors. Three technical replicates were set for each stage.

### 2.4. Cluster Analysis of Differential Metabolites

Hierarchical cluster analysis (HCA) of samples and metabolites was visualized as heatmaps with dendrograms. For HCA, normalized metabolite intensities (unit variance scaling) were displayed using a color gradient. The metabolomic profiles from the four stages were subjected to pairwise comparisons. Metabolites with a false discovery rate (FDR) of <0.05 and absolute Log_2_FC (fold change) value > 1.0 in any comparison were defined as differentially metabolites (DEMs). A series test of cluster analysis was performed according to a similar accumulation pattern by OmicShare tools (OmicShare Biotechnology Co., Ltd., Guangzhou, China) [35].

### 2.5. Metabolite Annotation and KEGG Enrichment Analysis

Identified metabolites were annotated using the Kyoto Encyclopedia of Genes and Genomes (KEGG) compound database, after which the annotated metabolites were mapped to the KEGG Pathway database. Pathways containing significantly regulated metabolites were then subjected to metabolite set enrichment analysis, and their significance was assessed using hypergeometric test *p*-values [36]. Correlations between metabolic pathways were evaluated based on the number of shared metabolites. The metabolite correlation index was calculated using Pearson’s correlation coefficients derived from metabolite accumulation data with the OmicShare tools.

### 2.6. RNA Extraction and Transcriptome Sequencing

Total RNA from seed at four stages (S1–S4) of *P. ostii* was extracted using the RNAprep Pure Plant Kit (Tiangen, Beijing, China) following the manufacturer’s instructions. For each sample, 1 μg RNA was used as input for library preparation. Twelve transcriptome libraries were constructed using the NEBNext UltraTM RNA Library Prep Kit for Illumina (NEB, Ipswich, MA, USA) following the manufacturer’s recommendations. The resulting mRNA was purified from total RNA using poly-T oligo-attached magnetic beads, followed by cDNA synthesis and purification with the AMPure XP system (Beckman Coulter, Beverly, MA, USA). The libraries were sequenced on an Illumina NovaSeq platform (Illumina, San Diego, CA, USA) to generate 150 bp paired-end reads.

### 2.7. Sequencing Data Analysis and Gene Functional Annotation

Clean data were obtained by removing adapter-containing reads, reads with poly-N, and low-quality reads. Q20, Q30, GC content, and sequence duplication levels were calculated, and clean reads were mapped to the reference genome. Transcript assembly and identification of known and novel transcripts were performed using the StringTie Reference Annotation Based Transcript (RABT) method based on Hisat2 alignment results.

Gene function was annotated using the following databases: Nr (NCBI non-redundant protein sequences), Pfam (Protein families), KOG/COG (Clusters of Orthologous Groups), Swiss-Prot (manually curated protein sequences), KO (KEGG Orthologs), and GO (Gene Ontology).

### 2.8. Gene Expression Analysis

Gene expression levels were quantified as fragments per kilobase of transcript per million mapped fragments (FPKM). Differentially expressed genes (DEGs) were defined as those with adjusted *p*-value < 0.01 and fold change ≥ 2, as determined by DESeq2 [37]. KEGG enrichment analysis of DEGs was performed using KOBAS 3.0. PCA, Venn diagrams, and KEGG pathway enrichment heatmaps were generated using OmicShare tools. Correlations between KEGG pathways were assessed based on the number of DEGs shared per pathway. Heatmaps of gene expression levels were produced from FPKM data using the OmicShare tools.

### 2.9. Gene Co-Expression and Potential Regulatory Relationship Analysis

The weighted gene co-expression network analysis (WGCNA) was conducted on the Metware Cloud platform using the R package (version 4.2.2). The soft threshold power was set to 18, the minimum module size to 50 genes, and the module merging threshold to 0.25. A hierarchical clustering tree was generated based on gene expression correlations and subsequently partitioned into modules. The expression patterns and correlations of hub module genes were quantitatively evaluated using intramodular expression levels.

DEG sequences were blasted against the *Arabidopsis* genome in the STRING database to obtain predicted protein–protein interactions. Promoter regions comprising 2000 bp upstream of each DEG were used to identify upstream transcription factors through the PlantTFDB database. The resulting DEG relationships were visualized in Cytoscape v3.8.0.

### 2.10. Quantitative PCR (qPCR) Analysis

qPCR reactions were performed using SYBR Green Master Mix (Vazyme Biotech Co., Ltd., Najing, China) on a LightCycler 96 system (Roche, Basel, Switzerland) with three technical replicates. *PoTubulin* (*PoTUB*) served as the internal control [38]. The qPCR program consisted of 95 °C for 30 s, followed by 40 cycles at 95 °C for 5 s and 60 °C for 15 s. Relative expression levels were calculated using the 2^−ΔΔCt^ method [39]. Primer sequences are listed in Table S2.

### 2.11. Statistical Analysis

Statistical significance was assessed using SPSS v19.0. For data meeting the normality and homogeneity of variance assumptions, one-way ANOVA followed by Duncan’s multiple-comparison test was performed. For data violating the normality assumption, we employed the non-parametric Kruskal–Wallis H test as the omnibus test. Upon finding a significant result (*p* < 0.05), we proceeded to post hoc pairwise comparisons. Data in all figures are presented as mean ± standard error (SE).

## 3. Results

### 3.1. Changes in Morphology During P. ostii Seed Development

**Figure 1:**
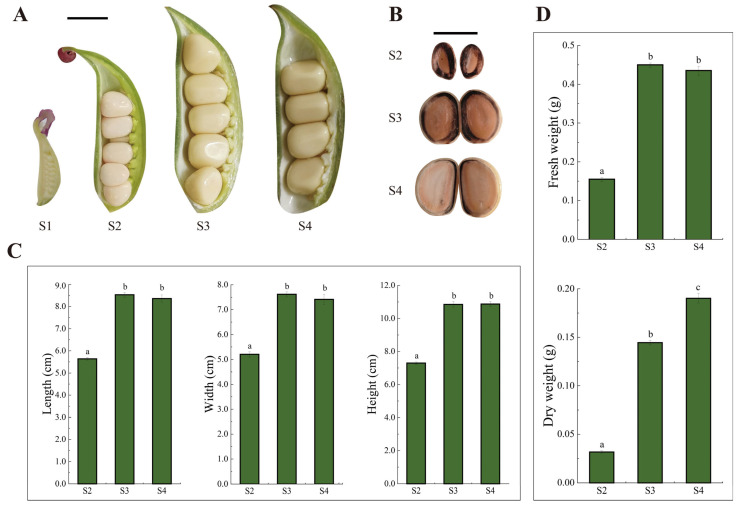
Morphological changes in *P. ostii* seeds at different developmental stages: (**A**) Dynamic changes in *P. ostii* fruit and seed development. (**B**) Starch staining intensity of the longitudinal section of the seed. (**C**) Length, width, and height of fresh seeds at different developmental stages. (**D**) Fresh weight and dry weight of seeds at different developmental stages. The scale bar in (**A**,**B**) is 1 cm. Lowercase letters a, b, and c indicate significant differences (*p* < 0.05).

Seeds at four developmental stages (S1, S2, S3, and S4) were collected, and their morphological progression was examined. Seed color gradually darkened, with S1 and S2 seeds remaining ivory white and those at S3 and S4 turning light yellow (Figure 1A). From S2 to S3, seed size increased markedly, from 5.64 ± 0.06 cm to 8.53 ± 0.16 cm in length, 5.02 ± 0.10 cm to 7.61 ± 0.10 cm in width, and 7.30 ± 0.07 cm to 10.85 ± 0.17 cm in height (Figure 1C, Table S3), reaching 8.36 ± 0.16 cm, 7.41 ± 0.20 cm and 10.87 ± 0.17 cm at S4, with no significant differences between S3 and S4. Fresh weight increased significantly at S3, whereas dry weight rose at both S3 and S4, peaking at S4 (Figure 1D). Although seed size remained similar between S3 and S4, starch staining intensity was stronger at S3 (Figure 1B).

### 3.2. Carbohydrate-Targeted Metabolites Content During P. ostii Seed Development

**Figure 2:**
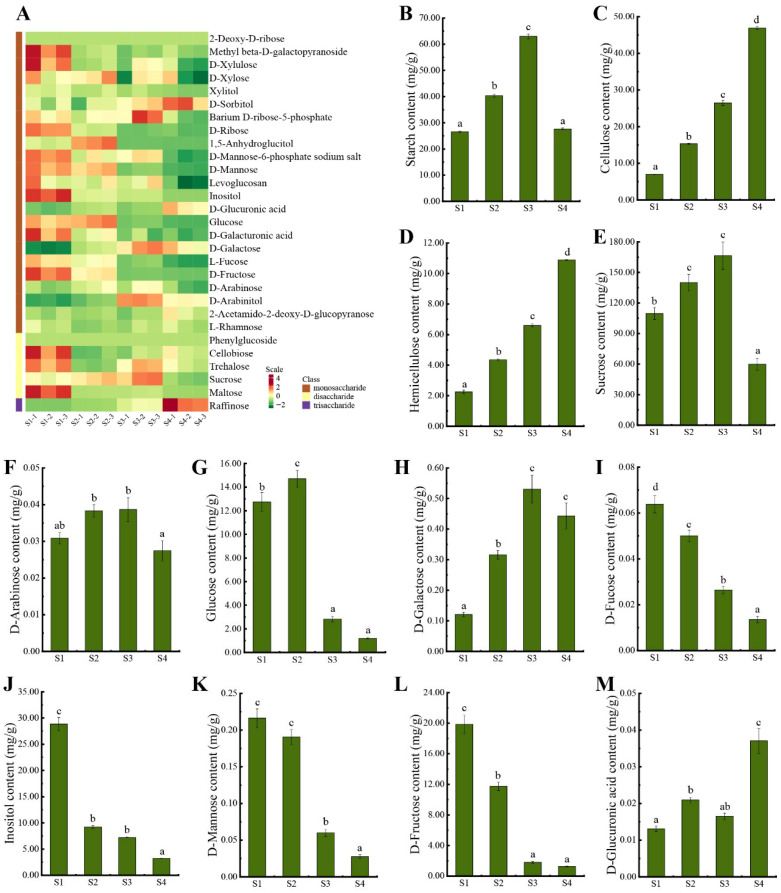
Metabolomics analysis of carbohydrate profiles in *P. ostii* seed at different developmental stages using GC-MS: (**A**) Hierarchical clustering analysis of the abundance of 27 carbohydrates. (**B**–**M**) The content of starch, cellulose, hemicellulose, sucrose, D-arabinose, glucose, D-galactose, D-fucose, inositol, D-mannose, D-fructose, and D-glucuronic acid. Red color indicates high content, while green indicates low content. Lowercase letters a, b, c, and d indicate significant differences (*p* < 0.05).

To characterize carbohydrate metabolism during seed development, carbohydrate-targeted metabolomic profiling was conducted across the four stages. Twenty-seven carbohydrate metabolites were identified by GC-MS and grouped into three categories: monosaccharides (22), disaccharides (4), and trisaccharides (1). Twelve monosaccharides and three disaccharides accumulated to higher levels at S1 than at later stages. Seven monosaccharides and one disaccharide peaked at S2 or S3, except 2-Ace-2-Deo-D-Glucosamine, Sorbitol, and Glucuronic-A, which showed maximum levels at S4. Raffinose, the sole trisaccharide detected, also peaked at S4. The starch, cellulose, and hemicellulose contents were quantified as major carbohydrate reserves. Starch content reached its maximum at S3 (Figure 2B), whereas cellulose and hemicellulose accumulated steadily from S1 to S4 (Figure 2C,D). These patterns indicate that many carbohydrates remain as free sugars rather than reserve compounds at S1. In S2 and S3, rapid seed growth and the synthesis of disaccharides and polysaccharides likely dilute monosaccharide levels. Several representative sugars were examined in detail. Increases in sucrose, starch, glucose, D-galactose, and D-arabinose (Figure 2E–H) and decreases in inositol, fructose, fucose, and mannose (Figure 2I–L) contents during S2 and S3 correspond to enhanced carbon assimilation and monosaccharide recycling. Structural polysaccharides (Figure 2C,D) and D-glucuronic acid (Figure 2M) were most abundant at S4, consistent with intensified cell wall deposition in the seed coat.

### 3.3. Selection of Metabolic Pathways Related to Key Carbohydrates in Seed

**Figure 3:**
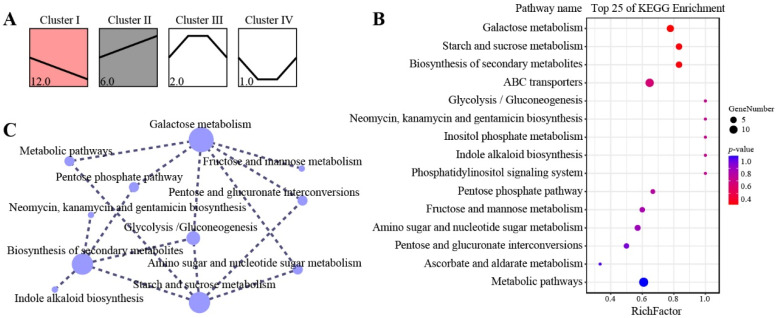
Trends in metabolite concentrations and functional analysis: (**A**) Series test of cluster analysis of differential metabolites (DEMs); the number represents the count of metabolites. (**B**) KEGG pathways enriched with DEMs. (**C**) Connectivity analysis among enriched metabolic pathways. Rich Factor represents the value of enrichment factor, which is the quotient of foreground value (the number of DEMs), and the larger the value, the more significant the enrichment. Coloring indicates *p*-value with higher in red and lower in blue, and the lower the *p*-value, the more significant the enrichment. Point size indicates the number of DEMs.

Carbohydrate dynamics across development were further assessed by screening differential metabolites (DEMs), resulting in 21 DEMs that were grouped into four clusters based on their abundance patterns (Figure 3A). Cluster I metabolites, including D-fructose, glucose, and inositol, were significantly downregulated (*p* < 0.05) as seeds matured. Cluster II metabolites, including D-glucuronic acid, raffinose, and structural polysaccharides, were significantly upregulated (*p* < 0.05). Cluster III, comprising sucrose and starch, increased and then decreased, with a peak at S3. In contrast, the Cluster IV metabolite cellobiose exhibited the opposite pattern and reached its lowest level at S2 (Table S4).

KEGG enrichment analysis showed that these metabolites were significantly enriched (*p* < 0.05) in galactose metabolism, starch and sucrose metabolism, and the biosynthesis of secondary metabolites (Figure 3B). Additional DEMs were detected in ABC transporters, general metabolic pathways, fructose and mannose metabolism, amino sugar and nucleotide sugar metabolism, and pentose and glucuronate interconversions. The distribution patterns of DEMs across these pathways revealed strong correlations among galactose metabolism, starch and sucrose metabolism, glycolysis, amino sugar and nucleotide sugar metabolism, and pentose and glucuronate interconversions (Figure 3C). These findings indicate that carbohydrate-related metabolic processes play a central role in determining seed size.

### 3.4. Metabolic Responses in Seeds at Different Development Stages Through the Pathways

**Figure 4:**
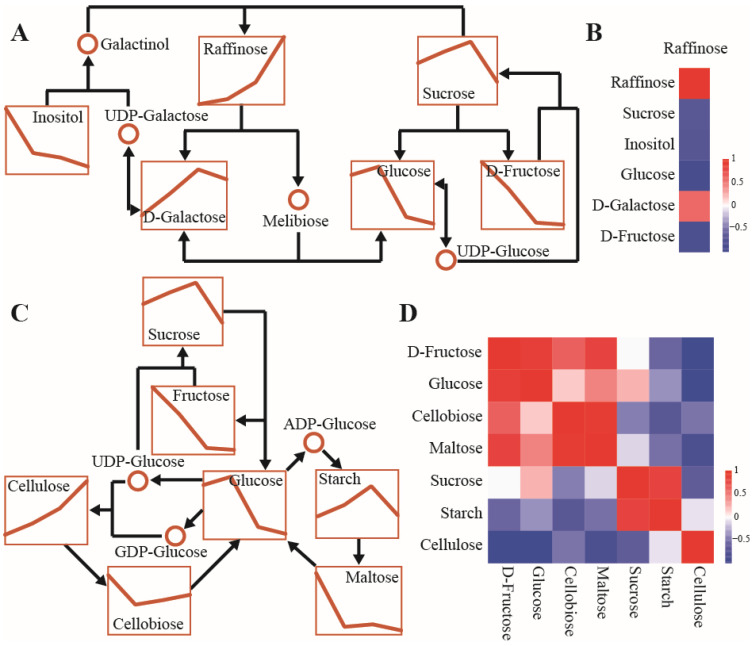
Change patterns in metabolite content and metabolic flux of the representative node in galactose, starch, and sucrose metabolism pathways: (**A**) Metabolite mapping on the galactose metabolic pathway with different developmental stages of seeds. (**B**) Heatmap indicating positive (red) and negative (blue) correlation between precursors and products of the galactose metabolic pathway based on Pearson’s correlation coefficient. (**C**) Metabolite mapping on the starch and sucrose metabolism pathway with different developmental stages of seeds. (**D**) Heatmap indicating positive (red) and negative (blue) correlation between precursors and products of the starch and sucrose metabolism pathway based on Pearson’s correlation coefficient.

Carbohydrate biosynthesis and degradation patterns were evident in metabolite changes across seed developmental stages. Mapping representative pathways (galactose metabolism, starch and sucrose metabolism, and amino and nucleotide sugar metabolism) allowed the identification of key metabolic fluxes. In the galactose metabolic pathway, raffinose content increased during seed development, which corresponded to significant decreases in upstream precursors (glucose, fructose, and inositol) due to substrate consumption. Sucrose, an intermediate metabolite, was strongly upregulated at S2 and S3 but declined at S4. Galactose, an isomer of glucose and a downstream product of raffinose degradation, increased initially and then decreased (Figure 4A). To clarify relationships among precursors, intermediates, and products, Pearson correlation analysis was conducted based on metabolite responses (Figure 4B). Most precursors were negatively correlated with their downstream intermediates and products, whereas galactose showed a positive correlation with raffinose.

The nucleotide-activated form of glucose serves as the precursor for sucrose, starch, and cellulose, creating competitive relationships among their biosynthetic pathways. Changes in metabolite levels associated with starch and sucrose metabolism indicated active synthesis of sucrose, starch, and cellulose at S2 and S3. However, sucrose and starch declined sharply at S4, while cellulose continued to increase (Figure 4C). Precursors and hydrolysis products were generally negatively correlated with these end products, and sucrose and starch were positively correlated with each other. In contrast, cellulose content exhibited opposite correlations with starch and sucrose, likely reflecting enhanced precursor allocation to cell wall formation at S4 (Figure 4D).

**Figure 5:**
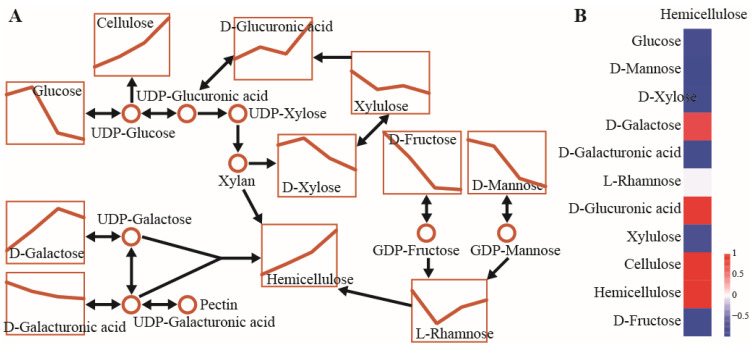
Change patterns in metabolite content and metabolic flux of the representative node in the amino and nucleotide sugar metabolism pathway: (**A**) Metabolite mapping on the amino and nucleotide sugar metabolism pathway with different developmental stages of seeds. (**B**) Heatmap indicating positive (red) and negative (blue) correlation between precursors and products of the amino and nucleotide sugar metabolism pathway based on Pearson’s correlation coefficient.

Several monosaccharides, including glucose, glucuronic acid, galactose, galacturonic acid, fructose, mannose, and xylose, participated in cell wall polysaccharide metabolism, and most showed significant downregulation. Glucuronic acid was strongly upregulated, while rhamnose decreased initially and then increased. Reduced glucose levels likely reflected interconversion to glycosyl compounds such as UDP-glucuronic acid, which contribute to cell wall remodeling through hemicellulose synthesis. Cellulose and hemicellulose contents were significantly upregulated, indicating active cell wall formation during seed development (Figure 5A). Correlation analysis between hemicellulose and its precursors or hydrolysis products showed strong positive correlations with galactose and glucuronic acid, whereas other monosaccharides were negatively correlated, consistent with dominant synthesis over hydrolysis reactions (Figure 5B).

### 3.5. The Transcriptome of P. ostii Seeds at Different Development Stages

To elucidate the molecular mechanisms underlying carbohydrate metabolism, twelve libraries from seeds collected at four developmental stages (S1, S2, S3, and S4) were sequenced. In total, 98.41 Gb of clean data were generated, with Q30 values exceeding 92.92% (Table S5), indicating high RNA quality. Clean reads were aligned to the *P. ostii* reference genome, yielding mapping rates of 81.92 to 84.49% (Table S6). A total of 79,002 genes were detected, including 66,027 known and 12,975 unknown genes. Of these, 25,452 genes displayed expression (FPKM ≥ 1) across all stages. Gene expression at S1 greatly exceeded that at later stages, reflecting elevated transcriptional activity as ovule tissues and specialized cell layers begin to differentiate (Figure S1A). PCA of the transcriptome data effectively separated the samples into four groups based on expression profiles, confirming their distinct developmental states (Figure S1B). Correlation analysis further clustered S1 and S2 together, and S3 and S4 together, consistent with the progression of seed development (Figure S1C).

### 3.6. Identification and Functional Enrichment Analysis of DEGs

**Figure 6:**
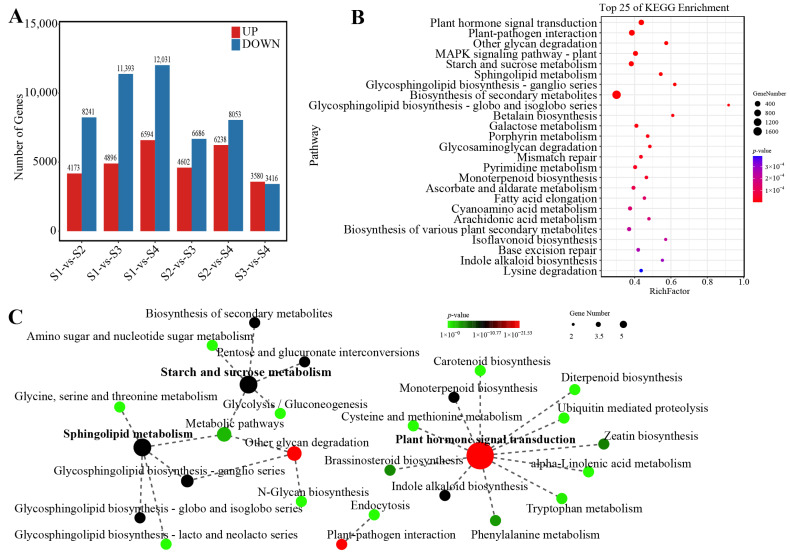
Identification and function analysis of DEGs: (**A**) Upregulated and downregulated DEGs in seeds between different development stages. (**B**) KEGG enrichment of the top 25 pathways based on DEGs. Rich Factor represents the value of enrichment factor, which is the quotient of foreground value (the number of DEGs), and the larger the value, the more significant the enrichment. Coloring indicates *p*-value with higher in red and lower in blue, and the lower the *p*-value, the more significant the enrichment. (**C**) Network of representative KEGG pathways. Coloring indicates *p*-value with higher in green and lower in red, and the lower the *p*-value, the more significant the enrichment. Point size indicates the number of DEGs.

Differential expression analysis revealed substantial temporal shifts. Relative to S1, 4173, 4896, and 6594 genes were upregulated, and 8241, 11,393, and 12,031 were downregulated at S2, S3, and S4, respectively (Figure 6A). More downregulated genes were observed, and the number of DEGs was reduced as the seeds matured. Overall, 26,092 DEGs were identified across four stages. KEGG enrichment assigned these DEGs to 140 pathways, with the top 25 shown in Figure 6B. Notably, 89, 282, and 93 DEGs were associated with other glycan degradation, starch and sucrose metabolism, and galactose metabolism, highlighting the central role of carbohydrate metabolism in *P. ostii* seed development. The most significantly enriched pathways (*p*-value < 1 × 10^−10^) included plant hormone signal transduction (ko04075), plant–pathogen interaction (ko04626), other glycan degradation (ko00511), MAPK signaling pathway (ko04016), starch and sucrose metabolism (ko00500), and sphingolipid metabolism. Pathway correlation grouped these processes into three clusters centered on plant hormone signaling, carbohydrate metabolism, including sphingolipid metabolism, and plant–pathogen interaction (Figure 6C). Integrating these patterns with metabolite dynamics, we infer that carbohydrate metabolism and biotic signaling represent major physiological activities during seed development.

### 3.7. Prediction of Hub Genes Associated with Carbohydrate Metabolism and Transport

**Figure 7:**
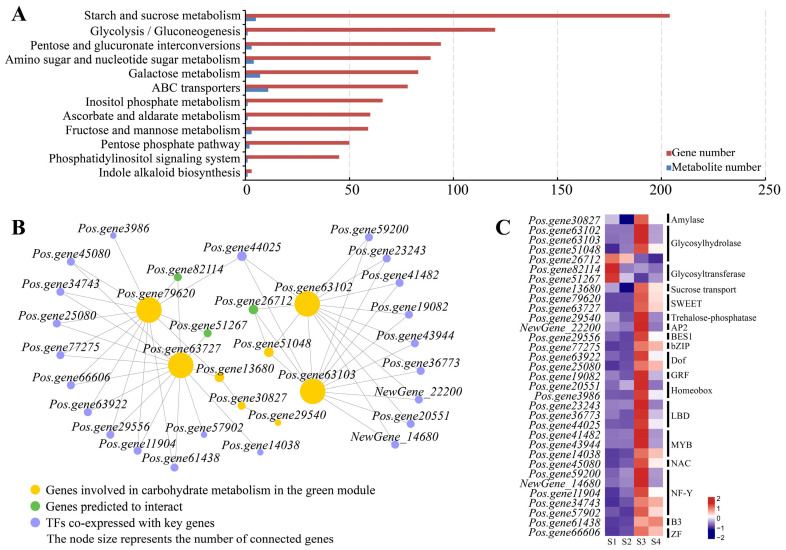
The conjugated analysis of carbohydrate metabolism and transport based on metabolome and transcriptome: (**A**) KEGG pathways co-enriched by DEGs and DEMs (**B**) Co-expressed network centered around genes encoding SWEET and glycosyl hydrolase. (**C**) Expression profiles of genes encoding structural genes and TFs in the co-expressed network. The color bar indicates log_2_-based fragments per kilobase per million (FPKM). The color indicates a high expression level in red and a low expression level in purple.

To identify candidate genes regulating carbohydrate metabolism and accumulation, a combined metabolomic and transcriptomic analysis was performed. The KEGG pathways co-enriched with DEGs and DEMs showed high DEG abundance in starch and sucrose metabolism, glycolysis/gluconeogenesis, and pentose and glucuronate interconversions, and high DEM abundance in ABC transporters, galactose metabolism, and starch and sucrose metabolism (Figure 7A).

DEGs involved in key carbohydrate metabolism and transport processes were subjected to co-expression and interaction prediction using weighted gene co-expression network analysis (WGCNA). The expression trend of DEGs in the green module, which peaks at stage S3, aligns with the patterns of sucrose and starch accumulation (Figure S2 and Figure 2B,E). Therefore, the green module was selected as the key module for hub-gene identification. There are 10 DEGs that involve in carbohydrate metabolism and transport processes and also belong to the green module, encoding three sugar transporters (*Pos.gene63727*, *Pos.gene79620*, and *Pos.gene13680*), four glycosyl hydrolases (*Pos.gene63102*, *Pos.gene63103*, *Pos.gene66338*, and *Pos.gene51048*), a UDP-sugar pyrophosphorylase (*NewGene_8538*), an alpha-amylase (*Pos.gene30827*), and a trehalose 6-phosphate phosphatase (*Pos.gene29540*). Protein–protein association analysis was performed for these 10 members, and their upstream transcription factors were predicted based on promoter sequences. A network comprising 33 genes was generated, with *Pos.gene63727*, *Pos.gene79620*, *Pos.gene63102*, and *Pos.gene63103* identified as hub genes (Figure 7B). The network includes 22 transcription factors from the NF-Y, MYB, LBD, Dof, and B3 families, as well as 11 structural genes. Most structural genes encode glycosyl hydrolases (*Pos.gene63102*, *Pos.gene63103*, *Pos.gene26712*, and *Pos.gene51048*), sugar transporters (*Pos.gene79620*, *Pos.gene63727*, and *Pos.gene13680*), or glycosyltransferases (*Pos.gene51267* and *Pos.gene82114*) (Table S7). According to the RNA-seq expression profiles, nearly all genes in the network peak at S3 or S4, except *Pos.gene26712*, *Pos.gene51267*, and *Pos.gene82114*, which are predicted to be co-expressed with the hub genes based on the STRING database (Figure 7C).

### 3.8. Expression Pattern Validation of the Hub DEGs and TFs

**Figure 8:**
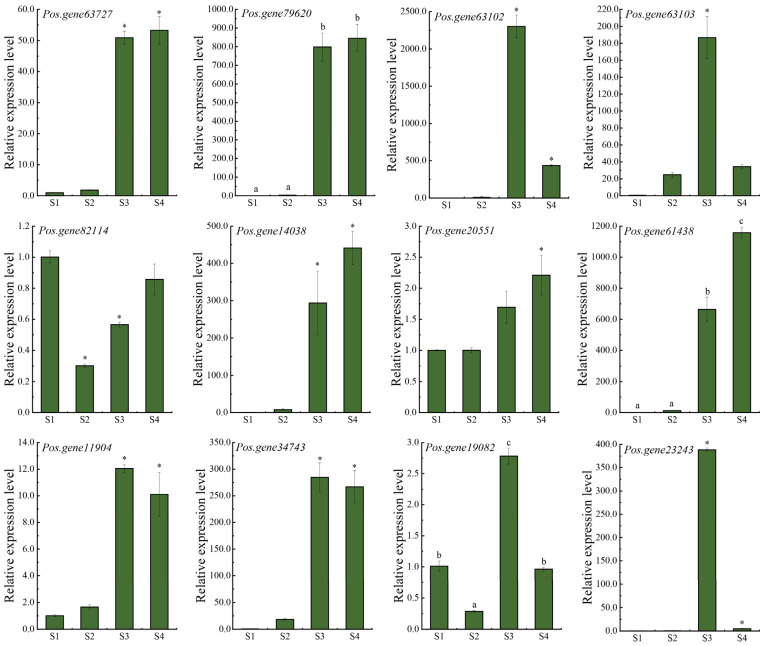
Expression patterns of the hub DEGs and TFs using quantitative real-time PCR (qPCR). Lowercase letters a, b, and c indicate significant differences (*p* < 0.05) based on Duncan’s test following one-way ANOVA, while Significant differences relative to stage S1 are marked with * (*p* < 0.05) based on the post hoc Dunn’s test following the Kruskal–Wallis analysis. The gene IDs correspond to the following proteins: sugar transporter SWEET (*Pos.gene63727* and *Pos.gene79620*), glycosylhydrolases (*Pos.gene63102* and *Pos.gene63103*), glycosyltransferase (*Pos.gene82114*), MYB (*Pos.gene14038*), Homeobox (*Pos.gene20551*), B3 (*Pos.gene61438*), NF-Y (*Pos.gene11904, Pos.gene34743*), GRF (*Pos.gene19082*), and LBD (*Pos.gene23243*).

To validate the RNA-seq data, representative DEGs from the co-expression network were selected for qPCR analysis across the four developmental stages of *P. ostii* seeds (Figure 8). The four hub DEGs were significantly upregulated at S3. Among them, the two DEGs encoding sugar transporters, *Pos.gene63727* and *Pos.gene79620*, maintained relatively high expression at S4, whereas the expression of *Pos.gene63102* and *Pos.gene63103*, which encode glycosyl hydrolases, decreased significantly in S4 but remained higher than at S1. *Pos.gene82114*, a glycosyltransferase potentially associated with sugar transporters in the co-expression network, showed high expression at S1 and was downregulated and then upregulated after pollination. These results were consistent with the RNA-seq profiles. To further identify potential regulators of the key genes, we examined the expression of TFs that were highly connected to the hub sugar transporter and glycosyl hydrolase genes. Their expression increased markedly from S2 to S3, in parallel to both the hub-gene expression patterns and sucrose and starch accumulation. Subsequently, TF expression patterns were grouped into three categories: sustained increase (*Pos.gene14038*, *Pos.gene20551*, and *Pos.gene61438*), stable (*Pos.gene11904* and *Pos.gene34743*), and decrease (*Pos.gene19082* and *Pos.gene23243*). Among these, *Pos.gene61438* exhibited the strongest upregulation at S3, followed by *Pos.gene14038*, *Pos.gene23243*, and *Pos.gene34743*, corresponding to members of the B3, MYB, LBD, and NF-Y families, respectively. Overall, qPCR confirmed that expression trends within the potential regulatory network were consistent with RNA-seq data and demonstrated that hub-gene and TF expression patterns are associated with sucrose and starch accumulation during seed development.

## 4. Discussion

Seed development influences major economic traits such as seed size and yield in oilseed crops, and carbohydrate metabolism is a key factor underlying these processes. Rapid post-fertilization seed expansion directly affects final seed size and yield, and this expansion is primarily driven by the rapid accumulation of carbohydrates during the grain-filling stage [40]. Seeds of soybean, oilseed rape, and oil tea are predominantly oil-producing. Analyses of their seed development show that sucrose and starch accumulate mainly during the middle or early developmental stages, which coincides with the period of rapid seed enlargement [8,41]. This pattern is consistent with our results, where sucrose and starch levels peaked at the S3 stage, the point at which seeds reached their maximum size, indicating that their accumulation is a major contributor to seed size. As seeds matured at the S4 stage, sucrose and starch contents declined, and the seeds became darker, likely associated with the biosynthesis of secondary metabolites such as fatty acids and flavonoids [42]. The reduced starch-staining area and enlarged cotyledons at S4 suggest that starch was partially hydrolyzed and used as a substrate for fatty acid synthesis. Previous studies reported that soluble sugar, starch, and crude fatty acid contents in peony seeds peaked at 60, 80, and 100 days after pollination, respectively, before declining, consistent with the overall trend observed here [5]. Minor differences in peak timing may result from variation in seed developmental rates linked to sampling year or growth conditions.

Uncovering the patterns of synthesis, hydrolysis, and transformation of carbon-based compounds is essential for understanding the molecular mechanisms governing carbohydrate accumulation. In this study, the 21 DEMs were categorized into three groups based on their variation patterns. Monosaccharides such as glucose, fructose, and inositol belonged to Cluster I, showing consistently decreasing levels, whereas sucrose, starch, raffinose, and structural polysaccharides increased or increased before decreasing at the S4 stage. In soybean, glucose and fructose accumulate during the early seed developmental period (R5) and decrease by R5.5, while sucrose and starch increase during R6 and decline in R7 [3]. This similar trend of content changes of different metabolites as observed in this study implied that the glucose and fructose during early seed development are primarily derived from the cleavage of sucrose by invertase and are not yet substantially utilized for fixed carbon storage biosynthesis, whereas with starch and sucrose metabolism, glucose and fructose are consumed as substrates for sucrose and starch synthesis, contributing to an increase in sucrose and starch content. In black beans, monosaccharides and disaccharides decrease during maturation, while certain oligosaccharides such as raffinose and stachyose gradually increase [42]. Similarly, we observed a significant increase in raffinose during seed development, accompanied by a substantial decrease in inositol, indicating that inositol serves as a substrate for raffinose synthesis and highlighting the regulatory role of raffinose in maintaining seed physiological activity [43,44]. Among the monosaccharides, 2-Ace-2-Deo-D-Glucosamine and D-Glucuronic-A were most abundant at the S4 stage. 2-Ace-2-Deo-D-Glucosamine is a major precursor for chitin, glycosylated proteins, and lipids [45], whereas D-Glucuronic-A is a key glycosyl donor for hemicellulose synthesis, contributing to xylan side-chain formation and interacting with phenolic and aliphatic compounds to form composite materials [46]. Thus, their elevated levels at S4 likely relate to the synthesis of these structural substances, consistent with the higher levels of cellulose and hemicellulose detected at the same stage.

This study quantified sugar-targeting metabolic patterns during peony seed development, providing a basis for screening and characterizing genes involved in these pathways and clarifying their roles in seed development and yield formation. The DEGs identified across developmental stages were mainly enriched in hormone signaling, carbohydrate metabolism, and secondary metabolite pathways, consistent with gene expression and functional enrichment patterns reported for soybean, grape, and buckwheat seed development [47]. In soybean and grape, comparative transcriptome analyses further showed that DEGs influencing seed size were concentrated in plant hormone and signal transduction, starch and sucrose metabolism, and flavone and flavonol biosynthesis pathways, reinforcing the view that seed expansion precedes substantial fatty acid synthesis [48,49]. In the present study, genes enriched in hormone signaling transduction were associated with auxin and cytokinin synthesis or degradation. These genes contribute to successful seed development through endosperm formation and cell proliferation and may be regulated by sucrose signaling [50].

Carbohydrate metabolism is a key determinant of seed development, particularly seed size. Here, changes in gene expression and metabolite content across developmental stages indicate that starch and sucrose metabolism dominate this process. Several candidate genes regulating starch accumulation were identified during the critical developmental period of *Castanea henryi* seeds, including three ADP-glucose pyrophosphorylase genes, one granule-bound starch synthase gene, and two beta-amylase genes [51]. During *Camellia drupifera* fruit development, genes most strongly correlated with sucrose content encoded sucrose phosphate synthase, sucrose synthase, sucrose transporters, and SWEET proteins [7]. In the present study, genes involved in sugar metabolism or transport were screened within co-expressed modules that tracked starch and sucrose content. The most highly connected genes encoded glycosyl hydrolases, sugar transporters, UDP-sugar pyrophosphorylase, alpha-amylase, and trehalose–phosphatase. These genes have recognized roles in carbohydrate metabolism and sugar accumulation during seed development, and sugar transporters in particular are essential for enhancing seed yield by optimizing carbon allocation.

Recent studies underscore the central role of sugar transporters in coordinating sugar and oil accumulation in seeds. In oil tea, *CoSUT4* is strongly expressed during seed development and enhances seed yield and oil content through sucrose transport [52]. In *Arabidopsis*, the *sweet11–sweet12–sweet15* triple mutant disrupts sugar movement from the seed coat to the embryo, causing severe seed developmental defects and reduced seed weight, starch, and oil content [20]. In soybean, *GmSWEET10a*, *GmSWEET10b*, and *GmSWEET15* facilitate sugar transfer from the seed coat to the embryo. Among these, *GmSWEET10a* underwent strong domestication selection, contributing to increased seed size and oil content [21,53]. Likewise, during oil tea seed development, *CoSWEET1b*, *CoSWEET2a*, and *CoSWEET15* participate in sugar transport to the embryo and endosperm [41]. GhSWEET42 is highly expressed during cotton seed development and enhances seed oil content through sugar transport [54]. In our co-expression network, two hub genes, *Pos.gene63727* and *Pos.gene79620*, encoded SWEET transporters and showed high expression during the period of rapid sucrose and starch accumulation. These patterns suggest potential functions in sugar transport during seed development. Further investigation is required to determine whether these genes enhance seed size, yield, and oil content in tree peony by promoting seed filling.

The genes encoding NF-Y, MYB, LBD, Dof, and B3 transcription factors were co-expressed with hub genes involved in sugar metabolism and transport, indicating that these transcription factors may also contribute to peony seed development. NF-Y and B3 TFs have similarly been implicated in seed development in other plant species [55]. In wheat, *TaNF-YA3-D*, *TaNF-YB7-B*, and *TaNF-YC6-B* are strongly expressed in the endosperm during grain filling. These TFs form a nuclear factor Y trimeric complex that regulates cytosolic small ADP-glucose pyrophosphorylase 1a (*TacAGPS1a*), sucrose synthase 2 (*TaSuS2*), and other starch biosynthesis genes, thereby promoting starch accumulation [56]. Within the NF-Y and B3 families, LEC1 and the B3 regulators ABI3, FUS3, and LEC2, collectively known as LAFL, are key determinants of seed development, endosperm formation, and oil deposition [57]. For example, CsFUS3 activates the *CsAGA2* gene encoding alkaline α-galactosidase 2, providing the sugars required for seed filling and regulating embryo development in cucumber [31]. In *Paeonia rockii*, *LAFL* genes are hypothesized to influence seed development and oil accumulation through *PrNF-YC2* activation, as both oil content and LAFL expression decline when *PrNF-YC2* is silenced [58]. In this study, several NF-Y and B3 genes were identified in the co-expression network, including *Pos.gene34743* (*LEC1*), *Pos.gene59200* (*LEC1-like*), and *Pos.gene61438* (*FUS3*), all belonging to the *LAFL* group. *Pos.gene59200* showed high expression at S3 of seed development, whereas *Pos.gene34743* and *Pos.gene61438* were significantly upregulated at S3 and maintained elevated levels at S4. These patterns suggest that both genes may contribute to seed sugar accumulation and oil synthesis. The homologs of *LEC1*, *LEC1-like*, and *FUS3* in Arabidopsis are also specifically and highly expressed in seeds, further supporting their essential roles in seed development [59,60]. Although most oilseed research has emphasized lipid biosynthesis, elucidating sugar accumulation during seed filling is vital for understanding seed size regulation and the metabolic substrates that support lipid synthesis. Integrating sugar metabolism genes with key transcriptional regulators of seed development may therefore provide a promising direction for future breeding strategies.

## 5. Conclusions

In this study, we analyzed the morphology, carbohydrate-targeted metabolome, and transcriptome of *P. ostii* seeds across developmental stages S1 to S4. Seed color gradually deepened during development, reaching maximum size at S3, coinciding with the highest sucrose and starch content. A total of 27 carbohydrate metabolites were detected. Most monosaccharides declined during seed development, whereas disaccharides and polysaccharides showed significant upregulation at S3 or S4. DEMs were predominantly enriched in galactose metabolism and starch and sucrose metabolism pathways. Metabolic flux analysis of representative pathways suggested that the reduction in monosaccharides and disaccharides may reflect their conversion into polysaccharides and other storage compounds. Transcriptome analysis identified 26,092 genes differentially expressed during seed development. Many DEGs were enriched in plant hormone signal transduction and starch and sucrose metabolism pathways. Integrative analysis of the metabolome and transcriptome revealed that KEGG pathways co-enriched with DEGs and DEMs include starch and sucrose metabolism, galactose metabolism, and ABC transporter pathways. Based on this, we constructed a potential regulatory network comprising 33 genes involved in sucrose and starch accumulation in seeds, and validated their expression patterns at different developmental stages via qPCR. Our results suggest that sugar transporters (SWEET and SUS), glycosyltransferases, and TFs, including NF-Y, MYB, LBD, Dof, and B3, play central roles in regulating seed sugar accumulation and development. This study provides a theoretical framework for improving the yield and quality of tree peony and lays the foundation for future efforts to manipulate genes involved in sugar allocation and distribution to enhance seed and oil production.

## Data Availability

The original contributions presented in the study are included in the article; further inquiries can be directed to the corresponding author.

## Supplementary Materials

The following supporting information can be downloaded at: https://www.mdpi.com/article/10.3390/biom16010017/s1, Figure S1: Overview of gene expression in *P. ostii* seed at four development stages. Figure S2: Correlation between module and samples based on WGCNA analysis. The color indicates positive correlation in red and negative correlation in blue based on Pearson’s correlation coefficient; Table S1: Information of authentical sugar standards used for GC-MS analysis; Table S2: Primers used for qPCR; Table S3: The width, length and height of seeds; Table S4: Statistics of differential metabolite sets based on metabolites content; Table S5: Sequencing data Statistics; Table S6: Transcriptome sequencing data mapping statistics; Table S7: Annotation of genes in co-expression networks.
