# Supplementary material for: Transcriptome and Metabolome Analyses Reveal the Accumulation Mechanism of Carbohydrates During Paeonia ostii Seed Development
Source: Biomolecules. 2025 Dec 22;16(1):17. doi: 10.3390/biom16010017 (PMC12838947; doi:10.3390/biom16010017)
Supplement: Supplementary file 1 [file biomolecules-16-00017-s001.zip › Figure S1-S2.pdf]

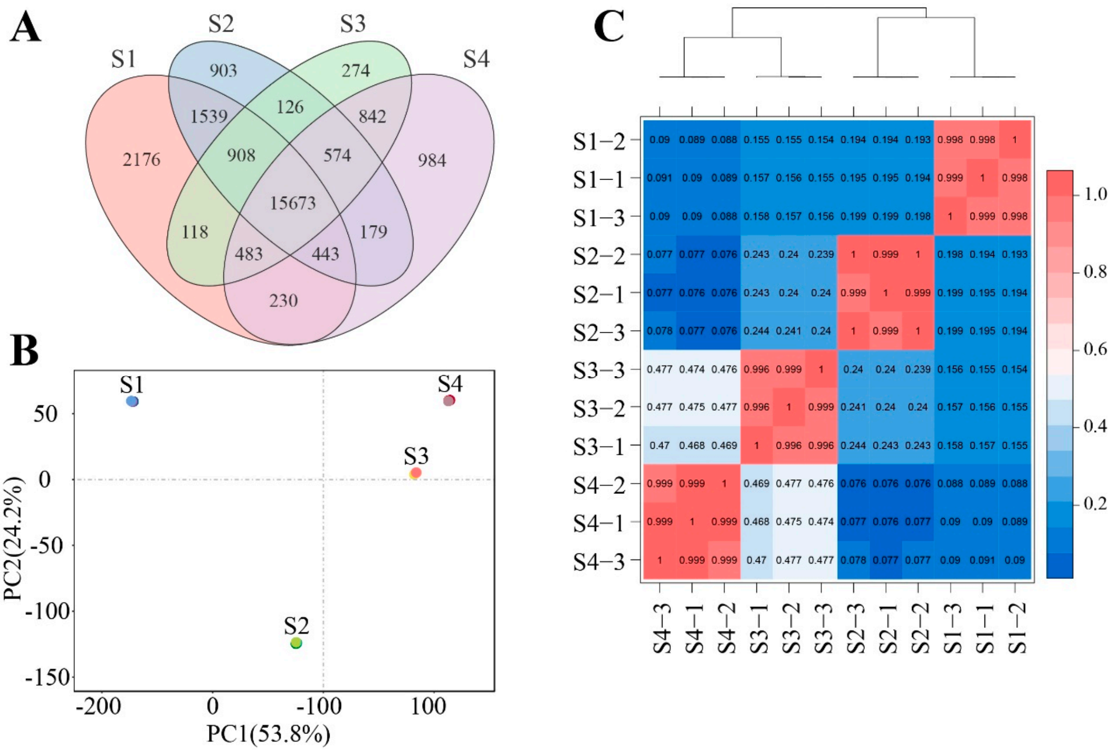

Figure S1. Overview of gene expression in *P. ostii* seed at four development stages. (A) Venn diagram of gene expression. (B) PCA analysis of twelve libraries from seeds. (C) Correlation of RNA-seq data among 12 samples. The color indicates positive correlation in red and negative correlation in blue based on Pearson's correlation coefficient.

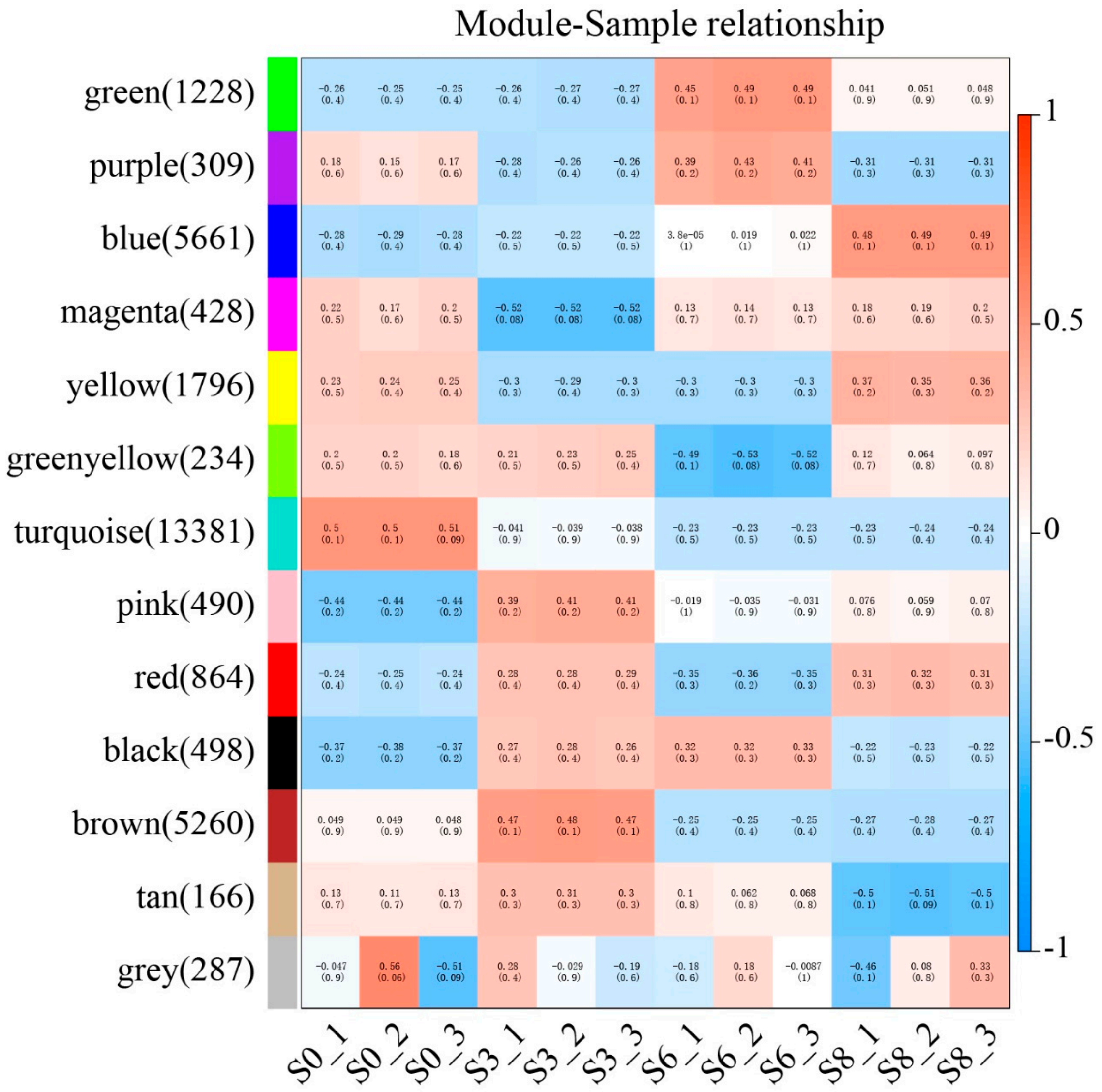

Figure S2. Correlation between module and samples based on WGCNA analysis. The color indicates positive correlation in red and negative correlationr in blue based on Pearson's correlation coefficient.
